# AI delivers Michaelis constants as fuel for genome-scale metabolic models

**DOI:** 10.1371/journal.pbio.3001415

**Published:** 2021-10-20

**Authors:** Albert A. Antolin, Marta Cascante

**Affiliations:** 1 Department of Data Science, The Institute of Cancer Research, London, United Kingdom; 2 Division of Cancer Therapeutics, The Institute of Cancer Research, London, United Kingdom; 3 Department of Biochemistry and Molecular Biomedicine & Institute of Biomedicine of Universitat de Barcelona, Faculty of Biology, Universitat de Barcelona, Barcelona, Spain; 4 Centro de Investigación Biomédica en Red de Enfermedades Hepáticas y Digestivas (CIBEREHD) and Metabolomics node at Spanish National Bioinformatics Institute (INB-ISCIII-ES-ELIXIR), Instituto de Salud Carlos III (ISCIII), Madrid, Spain

## Abstract

Michaelis constants (K_m_) are essential to predict the catalytic rate of enzymes, but are not widely available. A new study in *PLOS Biology* uses artificial intelligence (AI) to accurately predict K_m_ on a proteome-wide scale, paving the way for dynamic, genome-wide modeling of metabolism.

The Michaelis–Menten equation was derived by Leonor Michaelis and Maud Menten to quantify the velocity of an enzymatic reaction using measurable concentrations of enzyme and substrate even before the exact nature of enzymes was elucidated (Fig 1) [1]. Despite the limitations, its broad applicability, simplicity, and elegance have made it a cornerstone of biochemistry over the last century [1].

**Fig 1:**
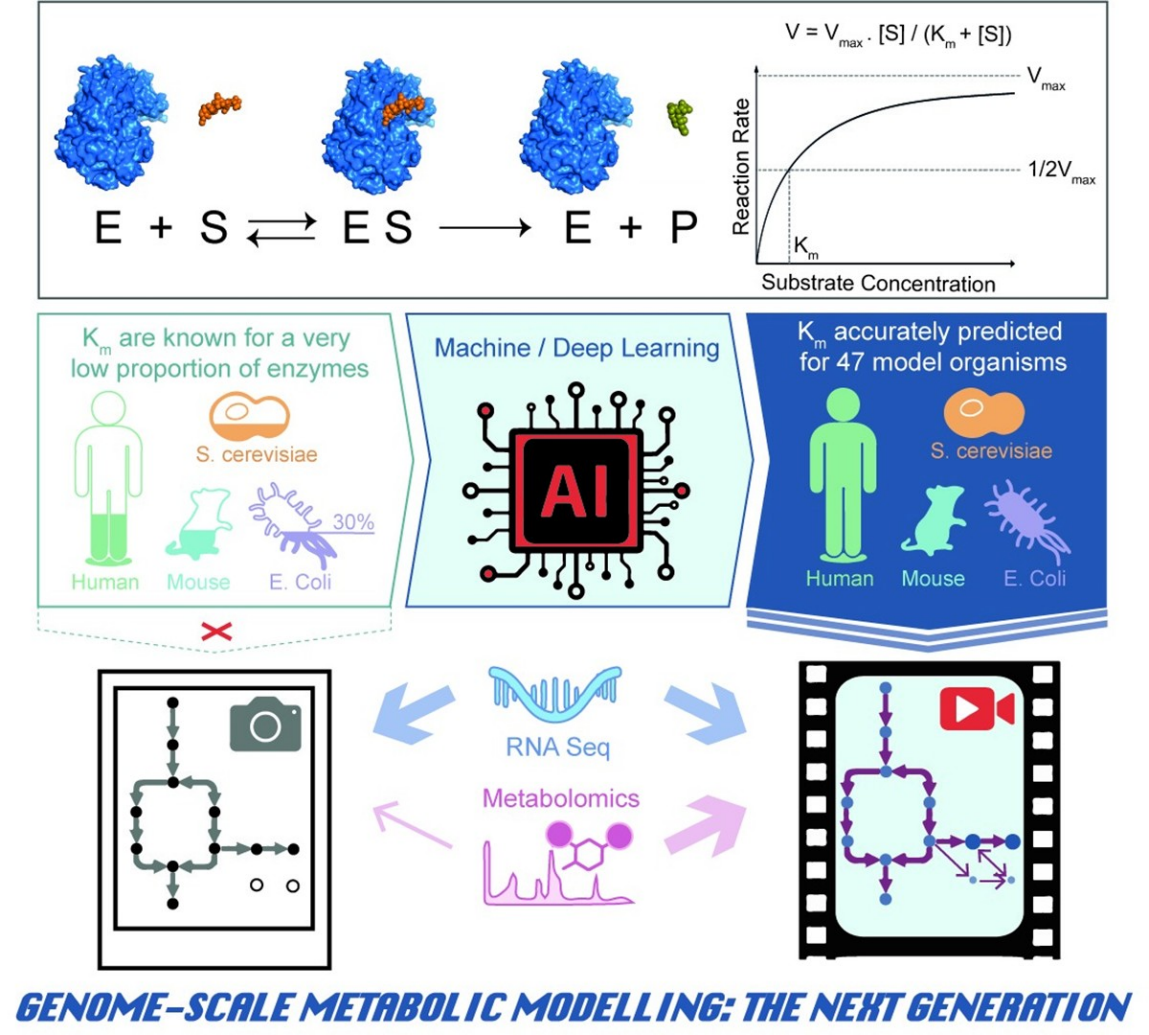
Impact of K_m_ availability on metabolic modeling. AI accurate and comprehensive prediction of K_m_ values, the key parameters related with enzyme substrate saturation, for 47 model organisms can be used to simulate dynamic metabolic flux changes at genome scale, facilitating the full exploitation of metabolomics data and opening new avenues in drug target discovery and metabolic engineering. AI, artificial intelligence; K_m_, Michaelis constant; RNA-seq, RNA sequencing.

The Michaelis constant (K_m_) in the equation is a pseudo-equilibrium constant that corresponds to the substrate concentration at which an enzyme operates at half of its maximum catalytic rate (Fig 1) [2]. Moreover, under certain assumptions, K_m_ is also an inverse measure of the affinity between the enzyme and its substrate [2]. K_m_ values can vary widely, often between 10^−1^ and 10^−7^ M [2]. Therefore, the determination of K_m_ is essential to predict catalytic rate of product formation and ideal substrate concentrations. This is important not only for fundamental research in enzymology but also for modern industrial biocatalysis, among other applications. Unfortunately, the experimental characterization of K_m_ values is laborious and time-consuming as it requires expressing and purifying enzymes and measuring their initial reaction rate at several substrate concentrations. Accordingly, K_m_ values in public repositories exist for only a small fraction of enzymatic reactions (Fig 1) [3]. For example, K_m_ values have been experimentally determined for less than 30% of *Escherichia coli*’s natural substrates (Fig 1)[3]. In turn, this lack of experimental data heavily limits its broad applicability in systems biology and metabolic modeling.

Artificial intelligence (AI), empowered by the increasing availability of Big Data, is transforming many aspects of our lives and multiple research fields [4]. Rooted in the 1950s, AI could be broadly defined as an algorithm that can “learn” patterns from training datasets and apply this learning to make new predictions [4]. We often subdivide the field between different types of “learning.” Machine learning (ML) uses hundreds of parameters that remain fully transparent to the researcher, but the ways in which they are combined are not always obvious. Deep learning (DL), in contrast, uses layered abstraction to identify key patterns in much more complex, sparse, and multidimensional data [4]. As recently illustrated by the impressive advances of Google’s DeepMind Alphafold2 in protein structure prediction, AI holds great potential to transform areas of research by releasing large-scale predictions that empower researchers worldwide [5]. Now, a new study published in *PLOS Biology* by Kroll and colleagues uses AI to predict K_m_ purely from protein and substrate information [6]. Their generalizable, organism-independent algorithm and predictions could have a transformative impact in several research fields.

The authors used K_m_ values from public databases to train AI models with an increasing amount of additional substrate and protein information [6]. First, they compared 4 different molecular fingerprints—vectors commonly used to numerically represent small molecules. Interestingly, a task-specific molecular fingerprint of the substrate generated using a graph neural network outperformed 3 traditional predefined molecular fingerprints. This result illustrates how DL can also be used to identify the best molecular representation [6]. The authors then compared a method of linear regression, a ML method and a DL method to train the models. Perhaps surprisingly, the ML method—gradient boosting—outperformed the other approaches, illustrating that more complex models are not necessarily better. Finally, the authors then used a cutting-edge deep numerical representation of the enzyme’s amino acid sequence, termed UniRep vector, to provide information on the enzyme. Interestingly, while the best model is the one using both enzyme and substrate information, the model only using substrate information outperforms the model only using enzyme information. The fact that the information on the exact residues comprising the catalytic site could not be provided probably contributes to explain this discrepancy, but it is interesting to speculate that this information is partially encoded in the substrate because the catalytic site has been optimized throughout evolution to fit the transition state of the substrate. The final model was appropriately validated using an independent dataset and predicted K_m_ values only deviated from experimental values by 4-fold on average. However, model performance was still increasing with the size of the training dataset, and, therefore, it will be important to continue improving the model as more experimental data become available, particularly regarding extreme values poorly represented in public datasets. Overall, Kroll and colleagues provide a very significant step forward that outperforms previous attempts at predicting K_m_.

Importantly, the authors not only provide the code in a public repository, but they also make available genome-scale K_m_ predictions for 47 model organisms. We foresee that these invaluable predictions will open new avenues of research in multiple fields. In particular, we think they could be an important step toward dynamic, genome-scale metabolic models (GSMMs). GSMMs emerged in the last decade as powerful constraint-based modeling platforms to achieve quantitative predictions of metabolic fluxes through multiomics data integration [7,8]. GSMMs have been successfully used for metabolic engineering and to identify cancer drug targets [9,10], but they are limited by the use of reconstructed metabolic reaction maps based on stoichiometric linear equations and pseudo steady state assumptions. One of the main bottlenecks is that kinetic parameters related to enzyme substrate saturation are not comprehensively available to be included in the equations describing enzyme reactions. This significantly limits model accuracy and provides a static model. A second, related, bottleneck is that metabolomics data can only be integrated qualitatively as metabolite concentrations cannot be calculated with current stoichiometric models. The deposition of K_m_ predictions proteome-wide by Kroll and colleagues could fuel a new generation of GSMMs that accurately predict dynamic metabolic flux maps to uncover new drug targets and boost our ability to quantitatively and accurately model metabolism.

## Abbreviations

AI: artificial intelligence
DL: deep learning
GSMM: genome-scale metabolic model
K_m_: Michaelis constants
ML: machine learning
